# Cases from Practice

**Published:** 1884-08

**Authors:** S. H. Dillard

**Affiliations:** Belton, Ga.


### CASES FROM PRACTICE.

BY S. H. DILLARD, M.D., BELLTON, GA.

CASE 1.—I was called to see Mrs. S., aged 30 years, on the morning of July 15th, 1883. I went immediately and found her in the second stage of labor, with fœtus in first cranial position, labor progressed nicely for half an hour, when the lady insisted on getting on her husband's lap. This I opposed but to no purpose. She got up and as she was in the act of sitting down we had a fracture of the femur at the junction of the middle and upper third of the bone. I then had her replaced in bed, and by the aid of the forceps, I delivered the child in fifteen minutes.

I then set the limb which had been broken, and left her until five o'clock in the afternoon when I returned, found her resting very well; temperature 99°, pulse 85.

The confinement previous to this, she had mammary abscess, it was lanced and after discharging for several days it healed, leaving induration of the entire gland. After her confinement on July 15th, 1883, the gland has diminished almost entirely, and has assumed a normal appearance. Her last child was well developed and lived to the age of five months.

The lady is of a scrofulous diathesis, and was unable to walk without the aid of crutches for three months before her confinement, on account of pain in the limb that was broken.

It was thought for quite a while before confinement that the trouble with the breast was malignant, and that it would require amputation. However, she recovered slowly, and is now seemingly as well as any one. She is able to attend to her household duties and walk four miles to town. Now, the question arises, what caused the fracture, and what caused the rapid diminution of the mammary gland that had remained so long indurated? Is it not a little surprising that the fracture should unite so readily in a person of scrofulous diathesis?

CASE 2.—I was called on the night of the 21st of April, 1883, to see Mrs. B., age eleven and a half years; found her in the first stage of labor, fœtus in second cranial position. The lady married at ten and a half years old. She had never menstruated up to the time of her marriage. She became pregnant very soon after marriage and was delivered of a well formed, healthy child at eleven and a half years old. So far as I know, this is the youngest mother on record.

